# Vitamin D level and endogenous DNA damage in patients with cancers in Duhok city, KRG-Iraq

**DOI:** 10.1016/j.amsu.2020.10.065

**Published:** 2020-11-10

**Authors:** Hishyar Azo Najeeb, Ramadhan Othman, Sherwan F. Salih, Ayad Ahmad Mohammed, Qais Al Ismaeel

**Affiliations:** a Department of Medical Chemistry, College of Medicine, University of Duhok, Kurdistan Region, Iraq; b Department of Internal Medicine, College of Medicine, University of Duhok, Kurdistan Region, Iraq; c Department of Surgery, College of Medicine, University of Duhok, Kurdistan Region, Iraq; d Department of Biology, Histology and Anatomy, College of Medicine, University of Duhok, Kurdistan Region, Iraq

**Keywords:** Cancer patients, Endogenous DNA damage, Plasma vitamin D, Vitamin D deficiency

## Abstract

**Introduction:**

Many clinical and pre-clinical studies suggested the protective effect of vitamin D against cancer development and cancer progression. Vitamin D deficiency is highly prevalent worldwide, and its link to DNA damage is worthy to study. It has been shown that vitamin D supplementation can reduce the risk of cancer with a favorable prognosis. Studies on DNA damage in different types of cancer and its link to plasma vitamin D has not been found in literature.

**Patients and methods:**

In this study we included 45 patients with different types of cancers and 35 healthy individuals as controls. The plasma vitamin D levels were measured in all participants. DNA damage levels of peripheral blood (mononuclear) cells in 45 newly diagnosed and untreated cancer patients and in 35 healthy individuals were measured using Alkaline Comet Assay technique.

**Results:**

The DNA damage observed in cancer patients was significantly higher than in healthy individuals. Interestingly, we have found a significant inverse correlation between the plasma levels of vitamin D and DNA damage in cancer patients (p < 0.0001) and in healthy individuals (p < 0.001).

**Conclusion:**

There is an inverse association between endogenous DNA damage and plasma vitamin D levels. Patients with vitamin D deficiency show highest levels of DNA damage suggesting that deficiency of vitamin D is probably one of the factors which increases the risk of cancer.

## Highlights

• **Vitamin D deficiency is highly prevalent worldwide, and its link to DNA damage is worthy to study.**
• **Transformation of normal tissue to neoplasms are the result of molecular changes in genetic materials.**
• **Deficiency of vitamin D probably one of the risk factors of cancer.**

## 1 Introduction

Exogenous and endogenous genotoxic factors constantly causing DNA damages in cells and cellular capability to eliminate these damages depends on the nature of DNA lesion and the efficiency of DNA repair mechanism. Any defect in the mechanisms of removing the damage could result in accumulation of genomic lesions within cells. Transformation of normal tissue to neoplasms are the result of molecular changes in genetic materials [[1], [2], [3]].

DNA damage refers to any change in the DNA structure physically or chemically. This damage effect can be raised by a variety of sources including exogenous and endogenous factors such as chemicals, radiation and free radicals. Genome instability has been found not only in tumor cells, but also in non-malignant cells of patients with cancer. Clinical and preclinical data showing an important role of DNA damage responses in cancer etiology, cancer development and its progression [[4], [5], [6], [7]].

Vitamin D deficiency is highly prevalent worldwide, and its association with DNA damage is potentially important. Studies on plasma vitamin D and DNA damage are few and data are conflicting. Most studies found a protective relationship between sufficient vitamin D status and lower risk of cancer. It has been reported that higher levels of plasma vitamin D, are substantially reduce the incidence rates of some cancers such as colon, breast, ovarian, renal, pancreatic, prostate and other cancers. Based on previous data that the supplementation of vitamin D can reduce the risk of some cancers including pancreatic and breast cancer [8,9].

A review article in a Harvard cohort study on vitamin D and the incidence of cancer reveals a protective relationship between sufficient vitamin D status and lower risk of cancer. Clinical prospective studies reported that higher levels of plasma vitamin D reduces the incidence rates of some cancers such as colon cancer, breast cancer and pancreatic cancer. Based on previous data of the Cohort, the supplementation of vitamin D can reduce the risk of some cancers including pancreatic and breast cancers. There is little data about the relation of excess vitamin D and cancer development [[9], [10], [11], [12]].

Further evidences show lower incidence and mortality rates from several cancers in regions with greater solar exposure. Experimental studies have also suggested a possible association between vitamin D and cancer risk. It has been reported that vitamin D play several activities that might slow or prevent the development of cancer, including promoting cellular differentiation, decreasing cancer cell growth and activating programmed cell death (apoptosis) [[3], [4], [5]].

The potential for vitamin D to reduce oxidative damage to DNA has been suggested by clinical trial where vitamin D supplementation reduced 8-hydroxy-2′-deoxyguanosine, a marker of oxidative damage, in colorectal cancer cells. However, data regarding the mechanism of protective effect of vitamin D against cancer development are still inconclusive. Thus, assessment of serum vitamin D and endogenous DNA damage in patients with different types of cancer could provide further evidence to its role in cancer prevention and progression. (6).

### 1.1 Patients and methods

A total number of 45 newly diagnosed and untreated patients (23 females and 22 males) with various types of cancers were enrolled in this study. All the patients were recruited from oncology section from Azadi Teaching Hospital, Duhok, Kurdistan Region, Iraq (Table 1). A group of 35 of healthy volunteers (age and sex were matched) contained 19 females and 16 males as control subjects were also included. The control individuals and patients were both none smoker and none taking food or vitamin D supplements. After signing the letter of consent by patients and participants, venous blood samples were collected into EDTA tubes and kept in deep freezer (−80) for DNA damage measurement. Enough amount of the same venous blood during sampling were placed into gel tubes without anticoagulants and the serum samples were prepared, marked and kept frozen for vitamin D measurement.

**Table-1:** Number of cancer patients and type of malignancy.

Type of Cancer	Female (n)	Male (n)
Gastric cancer	2	3
Colon cancer	2	4
Breast cancer	10	–
Thyroid cancer	1	–
Neuroendocrine cancer	1	–
Cervical cancer	2	–
Uterine cancer	2	–
None-small cell lung cancer	–	9
Bladder cancer	–	1
Ovarian cancer	3	–
Broncogenic carcinoma	–	2
Renal cancer	–	1
Hepatocellular carcinoma	–	1
Small cell lung cancer	–	1
**Total number**	**23**	**22**

### 1.2 Plasma vitamin D3 measurement

Circulatory form of plasma vitamin D3 for each patient and participant was measured by electrochemiluminescence immunoassay method using Cobas E−601. Vitamin D deficiency was considered when its plasma level less than 20 ng/ml.

### 1.3 DNA damage measurement

**Fig. 1:**
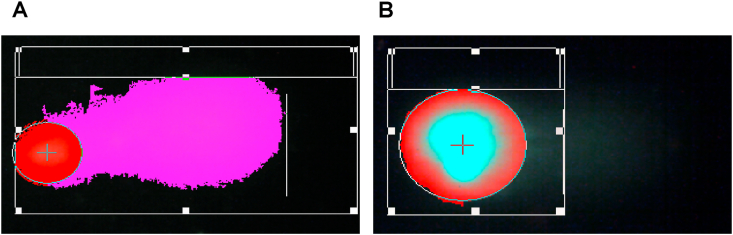
(A) Showing the migrated DNA tail (%Tail DNA) and the intact DNA (B) with no damage. The two captured DNA were analyzed by CASPLab software.

DNA damage was measured in peripheral blood cells **(mononuclear cells)** for all participants using Alkaline Comet Assay (ACA) technique at the Gentoxicity Research Unit/DMRC/College of Medicine/Duhok University. The ACA technique is also called single cell gel electrophoresis and considered as a very sensitive method for measurement of DNA damage at cellular level. Based on the previous studies [13]. The ACA was adapted to measure genomic damage in all participants enrolled in the present work. The assay was carried out through different steps starting from slide preparation and gel formation to electrophoresis and scoring the results. Briefly, 10 μL of whole blood was transferred into eppendorf tube and mixing with 180 μL of pre-wormed low melting point agaros (LMP) solution. Then 80 μl of mixture was dispensed to each half of a pre-coated microscopic slide (pre-coated with 1% normal melting point agarose). The two gels were covered directly with coverslips and allowed to solidify for 15 min one ice and protected from light. After that, the coverslips were removed and slides carefully transferred into pre-cold lysis buffer (100 mM disodium EDTA, 2.5 M NaCl, 10 mM Tris HCl, pH 10 containing 1% Triton-X-100 (v/v) in coapling jars and kept overnight at 4c. After lysis step the slides were washed once with ice-cold double distilled water (dH2O) followed by two more rinsing with ice-cold dH2O each left 20 min under dark position. After washing steps the slides were placed onto electrophoresis tank (Cleaver scientifica) and the ice-cold alkaline buffer solution (300 mM NaOH, 1 mM disodium EDTA, pH > 13) was added and left for 20 min allowing the embedded supercoil DNA to unwind. Following the incubation with alkaline buffer the electrophoresis was run for 20 min with a current 300 MA and 30 V A. After electrophoresis the slides were transferred into trays and washed with neutralizing buffer following by two wash with ice-cold dH2O. Slides were allowed to dry overnight and stained then with syber green dye (Thermo fisher scientific) before scoring and measurement of DNA damage. Each DNA score under fluoresce microscope showing head and tail of the DNA. The intact DNA with only a circle head means no damage Fig. 1B. The long migrated tail means the damaged amount in the DNA (%Tail DNA) Fig. 1A. During scoring, from each gel fifty comets were selected and captured for measurement using a fluorescence microscope (Leica fluorescence microscope) at 20× magnification.

### 1.4 Statistical analysis

The statistical analysis was performed using Graph Prism Pad software (Version 4.0). All data will be coded appropriately and the qualitative variables were given as frequency and percentage. Unpaired *t*-test was used to identify deviations in means between two groups. Regression analysis was performed to identify the effect between two different of variables. A significance level of p < 0.05 will be considered statistically significant.

### Ethical approval

The present study was approved by the scientific committee of the college of medicine/University of Duhok and obtained an ethical approval from general directorate of health of Duhok province-KRG-Iraq (**reference number: 18032017**–**3**). This study was conducted during (2018–2019).

The research is registered according the World Medical Association's Declaration of Helsinki 2013 at the research registry at the 19th of September 2020, Research registry UIN: research registry 6034.

The work of this article has been reported in line with the STROCSS criteria. [14].

## 2 Results

Table 1 shows the number of males and females with cancers included in the present study. This case controlled study involved 45 cases of newly diagnosed and untreated cancers (22 males and 23 males) and 35 healthy individuals (19 females and 16 males) were randomly selected. The age and sex matched between two groups and the difference in their age and body mass index (BMI) was not significant (Table 2).

**Table 2:** Demographics of study groups; results are expressed as Mean ± SD.

	Cancer Patients (45)	Healthy individuals [35]	*p*
**Age (years)**	**61.87 ± 12.77**	**58.5 ± 13.75**	**0.3298**
**BMI kg/M2**	**25.52 ± 3.802**	**24.38 ± 4.069**	**0.8211**

**Fig. 2:**
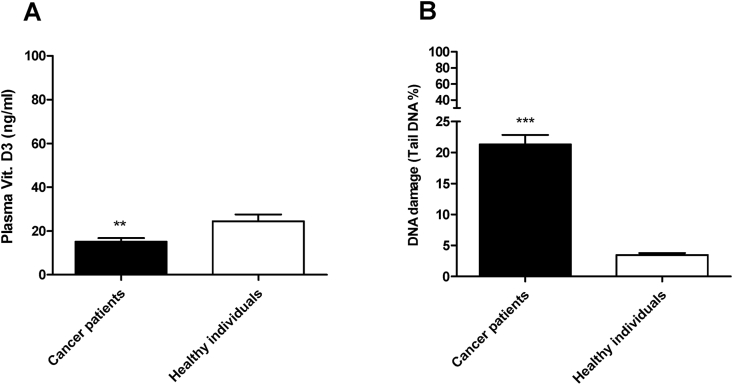
A) difference in the plasma level of Vitamin D3 between cancer patients and healthy individuals; data expressed as Mean ± SD. B) Difference in the mean of the DNA damage in the peripheral cells between cancer patients and healthy individuals. Data expressed as mean ± SE.

Serum samples of all study groups were first analyzed for the measurement of plasma vitamin D3 level. The majority of participants of both groups were vitamin D deficient. Since 57% of healthy individuals of this study with plasma vitamin D3 lower than 20 ng/ml and more than 82% of cancer patients with plasma vitamin D3 lower than 20 ng/ml. The plasma vitamin D3 in cancer patients significantly lower than that in healthy individuals. The mean ± SD of plasma vitamin D3 in healthy individuals and cancer patients were 24.43 ± 18.17 and 15.17 ± 11.11 respectively with a *p* value = 0.006 (Fig. 2A).

**Fig. 3:**
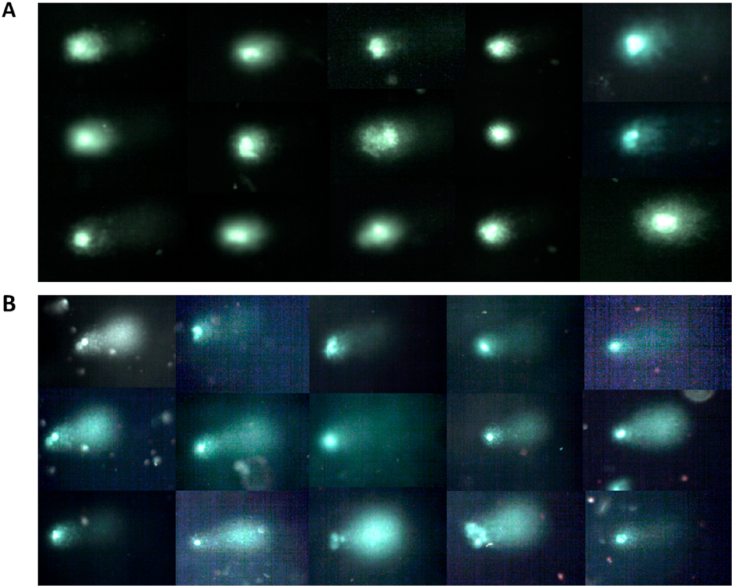
DNA damage of pictures peripheral blood cells captured by fluorescent microscope in healthy individuals (A) and in cancer patients (B).

**Fig. 4:**
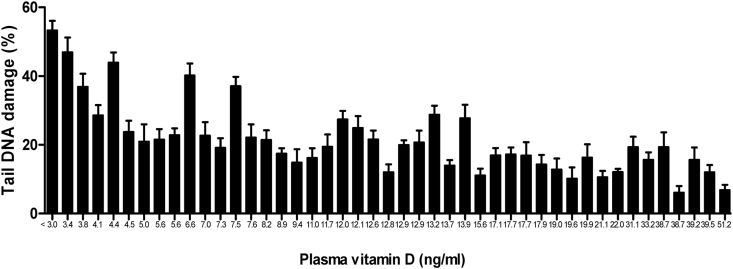
DNA damage levels measured by ACA in peripheral cells of patients with cancer and their plasma concentration of vitamin D3.

**Fig. 5:**
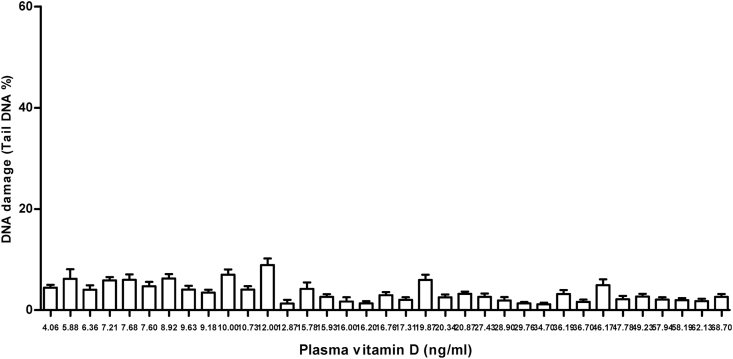
DNA damage levels measured by ACA in peripheral cells of healthy individuals and their plasma concentration of vitamin D3.

ACA experiments were run as planned in the materials and methods in this study and its data show higher levels of DNA damage in peripheral cells of cancer patients than the damage measured and observed in healthy individuals. The mean of DNA damage (Mean ± SD) in cancer patients was significantly higher than in healthy individuals, 21.31 ± 10.35 V s. 3.469 ± 1.952, respectively with a *p* value < 0.0001(Fig. 2B). Unlike in healthy individuals, greater DNA damages were observed in peripheral cells of most cancer patients particularly in those with advanced stage of the disease and those with lowest level of the plasma vitamin D. These genomic damages observed by fluorescent microscope presented with a very small head and huge amount of tail (Fig. 3). Interestingly our data showing that the levels of endogenous DNA damage in both groups decrease with the increase in the plasma concentration of vitamin D (Fig. 4 and Fig. 5). Furthermore, our data showing the DNA damage levels in patients with advanced stages of cancer (regardless of type of cancer) are significantly higher than the damage measured in those with earlier stages. The mean of DNA damage (% tail DNA) in patients with advanced and earlier stage of cancers were 27.84 ± 2.639 and 16.54 ± 1.196, respectively and with a p value = 0.0001.

**Fig. 6:**
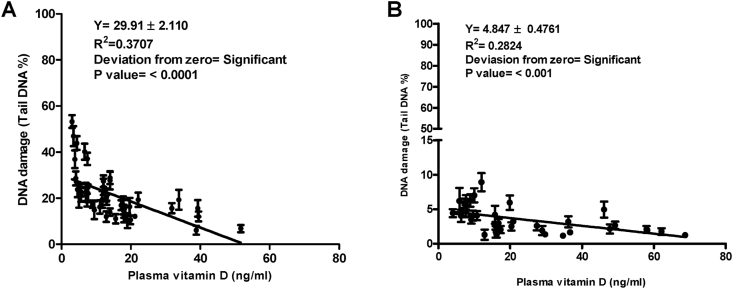
Relationship between DNA damage in peripheral blood cells and plasma levels of vitamin D. DNA damage levels *Vs*. plasma concentration of vitamin D3 in cancer patients (A) and in healthy individuals (B).

Furthermore, our data showing the DNA damage levels in patients with advanced stages of cancer (regardless of type of cancer) are significantly higher than the damage measured in those with earlier stages. The mean of DNA damage (% tail DNA) in patients with advanced and earlier stage of cancers were 27.84 ± 2.639 and 16.54 ± 1.196, respectively and with a p value = 0.0001. Likewise, the concentrations of plasma vitamin D in patients with advanced and earlier stages of cancers were 6.531 ± 2.501 and 21.48 ± 2.10, respectively and with a p value < 0.0001 (Table 3). In addition, our data have revealed a significant correlation between DNA damage in peripheral cells and plasma concentration of vitamin D in cancer patients and in healthy individuals. Association analysis showed an inverse relationship between the amount of damaged DNA and the concentration of plasma vitamin D in cancer patients with a p value < 0.0001 and in healthy individuals with a p value < 0.001 (Fig. 6).

**Table 3:** Concentrations of plasma vitamin D and the severity of DNA damage in patients with advanced and early stages of cancer.

Patients	Advanced stages (n = 19)	Early stages (n = 26)	P = value
DNA damage (% tail DNA) Mean ± SD	27.84 ± 11.50	16.54 ± 6.096	0.0001
Plasma Vitamin D3 (ng/ml) Mean ± SD	6.531 ± 2.501	21.48 ± 2.10	<0.0001

## 3 Discussion

Vitamin D deficiency is highly prevalent worldwide, and its association with DNA damage is potentially important. The link between circulatory vitamin D levels and cancer has been found by many epidemiological studies. Recent data indicate a significant association between low level of vitamin D and cancer prognosis. Previous data also reported a protective relationship between sufficient vitamin D status and lower risk of cancer. Since, it has been shown that the reduction in the vitamin D level is strongly linked to increased mortality rates from cancer. In addition, cancer incidence rates substantially reduced by sufficient amount of the main circulatory form of vitamin D, 25(OH) D [[15], [16], [17], [18], [19], [20], [21]].

Early epidemiological studies indicating vitamin D deficiency in women with breast cancer and showing an inverse relationship between the incidence and mortality rates of breast cancer and vitamin D status. In the present study we have found that most participants including cancer patients and healthy individuals were deficient in vitamin D. However, the mean value of plasma vitamin D3 in cancer patients was significantly lower than in healthy individuals. The mean ± SD of plasma vitamin D3 in healthy individuals and cancer patients were 24.43 ± 18.17 and 15.17 ± 11.11 respectively with a *p* value = 0.006. Clinical studies revealed a highly prevalence of vitamin D deficiency in newly diagnosed cancer patients**.** A very recent clinical study has also found insufficiency and deficiency of vitamin D in most newly diagnosed colorectal cancer patients [[22], [23], [24], [25]].

DNA damage in human cells is taken into consideration in cancer initiation and its progression. It has long been identified as an initial factor in malignant transformation. The damage formation in DNA of cancer patients has been reported by many studies. Accumulated damages in DNA not only comprises a root cause for cancer development but also continues to accelerate the cancer progression. It was suggested that cancer tissue of a patient contains a greater amount of DNA damages compared with normal tissue of the same patient. However, many other studies revealed that genomic damage not only in cancer cells but also in non-malignant cells, particularly in the peripheral blood cells, of the same patient [[26], [27], [28], [29], [30], [31]].

ACA data of the present study showing a greater amount of DNA damage in the peripheral blood cells of cancer patients compared to that in healthy individuals. The mean of DNA damage in cancer patients was significantly higher than in healthy individuals, 21.31 ± 1.51 V s. 3.469 ± 0.3299, respectively, (p < 0.0001). Among cancer patients a greater damage amount was found in those with advanced stages of the disease. The mean of DNA damage (% tail DNA) in patients with advanced and earlier stage of cancers were 27.84 ± 2.639 and 16.54 ± 1.196, respectively and with a p value = 0.0001. These data are consistent with the finding of a previous study showing higher level of background DNA damage in lymphocytes of patients with squamous cancer [32].

Buchynska et al. recently found more than 27% DNA damage in endometrial cancer cells and around 10% of DNA damage was observed in peripheral blood cells of those with endometrial cancer. Interestingly in both groups of study the DNA damage levels were higher in those with lower levels of plasma vitamin D and vice versa; but the effect was more sever in cancer patients. An experimental study showed more induced DNA damage in blood mononuclear cells in rats with vitamin D deficiency. Another experimental study has also showed a genotoxic effect of vitamin D deficiency. More interestingly, we have found a significant inverse relationship between plasma level of vitamin D and the amount of DNA damage in cancer patients and healthy individual. This relationship was more prominent in cancer patients (<0.0001) compared with that in healthy donors (<0.001). This might be the reason of the role of vitamin D to minimize the background DNA damage particularly in cancer patients [33,34].

Evidences in the literature are highly suggesting that vitamin D deficiency is associated with cancer risk and its progression. We have found an inverse association between endogenous DNA damage of peripheral blood cells and plasma vitamin D in cancer patients. Further clinical studies (with increased sample size) are needed to include a large number of different cancer patients; using vitamin D supplement then measuring endogenous DNA damage could further proof the role of vitamin D.

## Author contribution

Please specify the contribution of each author to the paper, e.g. study design, data collections, data analysis, and writing. Others, who have contributed in other ways should be listed as contributors. Dr Hishyar Azo Najeeb, Dr Sherwan F Salih, Dr Ramadhan Othman, and Qais Al Ismaeel did the data collection. Study design, analysis, and writing is done by Dr Hishyar Azo Najeeb and Dr Ayad Ahmad Mohammed. Final approval of the manuscript is done Dr Hishyar Azo Najeeb and Dr Ayad Ahmad Mohammed.

## Guarantor

The Guarantor is the one or more people who accept full responsibility for the work and/or the conduct of the study, had access to the data, and controlled the decision to publish.

## Funding

The author was only financial supporter of the study.

## Declaration of competing interest

There is no conflict of interest to be declared.
